# Association of Single-Nucleotide Polymorphisms in DC-SIGN with Nasopharyngeal Carcinoma Susceptibility

**DOI:** 10.1155/2017/6309754

**Published:** 2017-06-14

**Authors:** Sisi Li, Zhifang Lu, Mengwei Yao, Sisi Ning, Yuan Wu, Xunzhao Zhou, Changtao Zhong, Kui Yan, Ying Xie, Zhengbo Wei

**Affiliations:** ^1^Department of Head and Neck Tumor Surgery, Affiliated Tumor Hospital of Guangxi Medical University, Nanning, China; ^2^Graduate School of Guangxi Medical University, Nanning, China; ^3^Guangxi Key Laboratory for High-Incidence Tumor Prevention and Treatment, Experimental Center of Medical Science of Guangxi Medical University, Nanning, China

## Abstract

The aim of this study was to explore potential relationships of four single-nucleotide polymorphisms (SNPs) in the gene encoding dendritic cell-specific intercellular adhesion molecule 3-grabbing nonintegrin (DC-SIGN) with risk of nasopharyngeal carcinoma (NPC). The DC-SIGN SNPs rs7252229, rs4804803, rs2287886, and rs735240 were genotyped in 477 unrelated NPC patients and 561 cancer-free controls. At rs7252229, risk of NPC was significantly lower in individuals with GC (odds ratio [OR] 0.076, 95% confidence interval [CI] 0.008–0.690), GG (OR 0.056, 95%CI 0.006–0.487), or GC + GG (OR 0.059, 95%CI 0.007–0.515) than in individuals with the CC genotype, after adjusting for age, gender, smoking history, and EBV-VCA-IgA status. At rs4804803, risk of NPC was significantly higher in individuals with the genotype GG than in those with the genotype AA (adjusted OR 9.038, 95%CI 1.708–47.822). At rs735240, risk of NPC did not change significantly with genotypes AG, GG, or AG + GG after adjusting for age, gender, and smoking history. However, when data were also adjusted for EBV-VCA-IgA status, three genotypes emerged as associated with significantly higher risk of NPC than the AA genotype: AG (OR 2.976, 95%CI 1.123–7.888), GG (OR 3.314, 95%CI 1.274–8.622), or GG + AG (OR 3.191, 95%CI 1.237–8.230). Our results suggest that DC-SIGN SNPs rs7252229, rs4804803, and rs735240 may influence NPC risk in the Chinese population. The mechanisms mediating this risk require a further study.

## 1. Introduction

Nasopharyngeal carcinoma (NPC) is one of the most common malignant tumors in southern China, especially Guangdong and Guangxi provinces. In these endemic areas, annual NPC incidence reaches 20–30 per 100,000 [1]. The primary risk factors for NPC include Epstein-Barr virus (EBV) infection, environmental carcinogens, and certain ethnic backgrounds [2].

EBV infection is strongly associated with NPC pathogenesis [3], and approximately 98% of all NPC cases are EBV-related [4]. EBV may help drive NPC by encoding several oncogenic proteins, such as LMP1, which transform infected epithelial cells. In addition, the virus helps transformed cells evade host immune responses [5, 6]. One protein involved in this immune evasion may be dendritic cell-specific intercellular adhesion molecule 3-grabbing nonintegrin (DC-SIGN), a type II membrane protein of the C-type lectin receptor superfamily [7] that is encoded by CD209 on chromosome 19p13.3. Expressed mainly on the surface of immature dendritic cells (DCs), macrophages, and B lymphocytes, DC-SIGN recognizes and induces interactions with many pathogens, including bacteria, virus, and parasites [8–13]. DC-SIGN on immature DCs can bind carcinoembryonic antigen (CEA), inhibiting DC maturation and thereby inducing tolerance of tumor cells [9]. The SKBR3 breast carcinoma cell line can interact with DC-SIGN on macrophages to induce interleukin-10 secretion, contributing to an immunosuppressive environment [14].

Several single-nucleotide polymorphisms (SNPs) in the DC-SIGN gene have been associated with elevated risk of human diseases such as tuberculosis, dengue, AIDS, and cancer. For example, the SNP rs2287886 in the area of the promoter and SNP rs7248637 in the 3′-untranslated region may be associated with susceptibility to colorectal carcinoma [15]. The SNPs rs2287886, rs735240, and rs735239 correlated with NPC risk in a Cantonese population from Guangdong [16], while rs4804800 and rs7248637 correlated with risk in a North African population [10].

In the present study, we investigated the possible genetic associations of the DC-SIGN SNPs with NPC risk in a Chinese population in the NPC-endemic Guangxi province of China.

## 2. Methods

### 2.1. Study Participants

The study was approved by the Ethics Committee of Guangxi Medical University. Unrelated NPC patients who were diagnosed with NPC and treated between July 2012 and June 2015 at the Affiliated Tumor Hospital of Guangxi Medical University (Guangxi, China) were enrolled. In addition, 561 healthy controls were recruited among those undergoing routine physical examinations at the Affiliated Tumor Hospital and the First Affiliated Hospital of Guangxi Medical University. The selection criteria for the healthy controls included the following: (1) no individual history of malignant tumors, (2) undergoing a health examination during the same enrollment period as the NPC cases, and (3) a local resident of Guangxi province. All study participants provided written informed consent.

### 2.2. Blood Collection

Peripheral venous blood (3 mL) was drawn from all subjects into EDTA-containing tubes, and genomic DNA was isolated using the TGuide Blood Genomic DNA Kit (Tiangen, Beijing, China) according to the manufacturer's instructions. DNA quality was evaluated using agarose gel electrophoresis. Samples of verifiable quality were stored at −20°C.

### 2.3. Selection of SNPs

A total of 4 SNPs (rs7252229, rs2287886, rs4804803, and rs735240) within DC-SIGN were selected to the following analyses based on the following selection strategy: (1) Using the 1000 Genomes Browser (http://phase3browser.1000genomes.org/index.html) and the NCBI database (http://www.ncbi.nlm.nih.gov), SNP files of DC-SIGN were obtained for the Beijing Han Chinese (CHB) population. (2) Using Haploview 4.2 software, the SNP files were analyzed and the tagging SNPs which met the below criteria were selected: (i) minimum allele frequency (MAF) of greater than 5% in the CHB population; (ii) location within the coding region or the promoter region or the 3′- or 5′-untranslated regions (UTRs); and (iii) no strong linkage disequilibrium (LD; *r*^2^ < 0.80) with any other selected SNPs. (3) The potential function of the tagging SNPs was analyzed using SNP function prediction in the website http://snpinfo.niehs.nih.gov/snpinfo/snpfunc.htm. (4) We also forced the inclusion of some SNPs which did not fulfill the above selection criteria, for their functionality has been indicated in published studies. (5) Compatibility of selected SNPs through the above steps with the platform of MALDI-TOF mass spectrometry-based genotyping was analyzed by the BGI company (Beijing, China), which provided the SNP genotyping support. And the SNPs which were not technically compatible with the genotyping platform were excluded from the following formal genotyping.

### 2.4. Mass Spectrometry-Based Genotyping

The DC-SIGN SNPs rs7252229, rs4804803, rs2287886, and rs735240 were genotyped in all subjects as described [17] using allele-specific matrix-assisted laser desorption/ionization time-of-flight (MALDI-TOF) mass spectrometry, with support from BGI (Beijing, China). Fragments spanning the SNPs were amplified by PCR using primers designed with MassARRAY Assay Design 3.1 Software (Sequenom, San Diego, CA, USA). Amplifications were conducted in a 384-well ABI Veriti PCR System (Applied Biosystems) following the manufacturer's instructions. Amplification reactions (5 *μ*L) contained 4 *μ*L Master Mix and 1 *μ*L DNA (20 ng/*μ*L), and reaction conditions were as follows: 94°C for 5 min, followed by 45 cycles of 94°C for 20 sec, 56°C for 30 sec, and 72°C for 1 min. Alleles were analyzed using MassARRAY TYPER 4.0 software (Sequenom). Successful genotyping rates were 99.8% (560/561) in the control group for rs7252229, rs4804803, and rs735240, respectively, and were 99.8% (476/477) in the NPC group and 99.6% (559/561) in the control group for rs2287886.

### 2.5. Statistical Analysis

Data were analyzed using SPSS 17.0 (IBM, Armonk, NY, USA), and the threshold for significance was defined as two-sided *P* < 0.05. NPC patients and healthy controls were compared in terms of gender composition, smoking history, and the presence of immunoglobulin A against EBV capsid antigen (EBV-VCA-IgA) using the chi-squared test. The ages of patients and controls were compared using an unpaired *t*-test. Genotype distributions in healthy controls were compared against the predictions of Hardy-Weinberg equilibrium (HWE) using the Pearson chi-squared test. Genotype frequencies were compared between patients and controls using the chi-squared test. Possible associations between DC-SIGN SNP genotypes and risk of NPC were identified using logistic regression; odds ratios (ORs) with 95% confidence intervals (CIs) were calculated when appropriate. Unconditional multivariate logistic regression was used to calculate adjusted ORs and 95%CIs after adjusting for age, gender, smoking history, and EBV-VCA-IgA status, unless otherwise noted. The goal was to eliminate possible effects of these confounding factors in order to isolate the effects of each SNP on NPC risk.

## 3. Results

### 3.1. Characteristics of Patients and Controls

A total of 477 NPC patients and 561 healthy controls were included in our study. While the two groups were similar in age (*P* = 0.056), they differed significantly in gender composition (*P* < 0.001), smoking history (*P* < 0.001), and EBV-VCA-IgA status (*P* < 0.001; Table 1). Frequencies of each SNP genotype were in agreement with HWE in the control population: rs7252229, *P* = 0.307; rs4804803, *P* = 0.533; rs2287886, *P* = 0.331; and rs735240, *P* = 0.937.

### 3.2. Correlation of rs7252229 Polymorphism with Risk of NPC

Frequency of the genotype GG + GC at rs7252229 was significantly lower in NPC patients than in healthy controls (0.18% versus 1.68%), corresponding to an unadjusted OR of 0.105 (95%CI 0.013–0.842) and an adjusted OR of 0.059 (95%CI 0.007–0.515; Table 2). The genotypes GC and GG were also associated with lower risk than the genotype CC: the respective unadjusted ORs were 0.111 (95%CI 0.014–0.914) and 0.104 (95%CI 0.013–0.834); the corresponding adjusted ORs were 0.076 (0.008–0.690) and 0.056 (95%CI 0.006–0.488).

### 3.3. Correlation of rs2287886 Polymorphism with Risk of NPC

Relative to individuals with the genotype AA, individuals with the genotype AG or GG were at similar risk of NPC based on unadjusted and adjusted OR calculations (Table 3).

### 3.4. Correlation of rs4804803 Polymorphism with Risk of NPC

Individuals with the genotype GG were at significantly higher risk of NPC than those with the genotype AA (Table 4). The unadjusted OR was 4.800 (95%CI 1.014–22.732) and the adjusted OR was 9.038 (95%CI 1.708–47.822).

### 3.5. Correlation of rs735240 Polymorphism with Risk of NPC

Frequencies of genotypes AA, AG, and GG did not differ significantly between patients and controls before or after adjusting for age, gender, and smoking history. Interestingly, after controlling for EBV-VCA-IgA status, the genotypes GG, AG, and GG + AG emerged as conferring higher risk of NPC, with respective ORs of 2.976 (95%CI 1.123–7.888), 3.314 (95%CI 1.274–8.622), and 3.191 (95%CI 1.237–8.230) (Table 5). Next, we compared genotype frequencies between patients and controls in separate subgroups among all subjects negative for EBV-VCA-IgA and among all subjects positive for EBV-VCA-IgA. Among those negative for EBV-VCA-IgA, the genotypes GG, AG, and GG + AG were significantly more frequent among patients than controls, with respective ORs of 8.797 (95%CI 1.175–65.838), 2.077 (95%CI 1.055–4.087), and 8.710 (95%CI 1.168–64.940). Among subjects positive for EBV-VCA-IgA, in contrast, genotype frequencies were similar between patients and controls.

## 4. Discussion

Our data showed significantly lower frequency of the GC + GG genotypes at rs7252229 in NPC patients than in healthy controls, suggesting that the genotype CC increases risk of NPC. This appears to be the first report linking rs7252229 with risk of malignant disease. Another study linked the C allele at rs7252229 with elevated risk of invasive pulmonary aspergillosis [11]. How rs7252229 may contribute to NPC pathogenesis is unclear. Since rs7252229 lies within an intron in the DC-SIGN gene and it is predicted to be a binding site for some transcripts (based on http://snpinfo.niehs.nih.gov/cgi-bin/snpinfo/snpfunc.cgi), we predict that the genotype CC at rs7252229 may affect DC-SIGN expression and thereby overall activity. It is also possible that rs7252229 polymorphism affects humoral and cell-mediated immunity: in a study of healthy school children and young adults who received the measles vaccine, the genotype CC was associated with higher specific neutralizing antibody titers than the genotype GG among African-Americans, while the genotype CC was associated with lower interferon-*γ* levels than genotypes GG and GC among Caucasians [12]. Thus, it is plausible that this SNP might be associated with immunological abnormality in NPC patients as well as immune escape by EBV-infected NPC cells. This should be investigated in future studies.

Our data showed a significantly higher frequency of the genotype GG at rs4804803 in patients than in controls, indicating that this DC-SIGN SNP is associated with elevated NPC risk. Our results are inconsistent with those of studies in a Cantonese population [16] and a North African population [10] that found no relation between this SNP and NPC risk. Two possible explanations for this discrepancy are that (1) differences in genetic background and linkage disequilibrium between our population of mostly Zhuang minority subjects and the Cantonese subjects in the previous study [16] may mean different effects of DC-SIGN polymorphism on NPC risk and/or (2) DC-SIGN alleles are present at different frequencies across populations. Indeed, the frequency of the G allele in our control group was 8% (82/1038), much lower than the frequencies of approximately 26% reported for Caucasian and North African populations [10, 13]. How the SNP rs4804803 may influence NPC risk is uncertain. It is located only 214 bp upstream from the major Sp1-like binding region. We speculate that polymorphism in this region affects DC-SIGN gene transcription and thereby its expression [16].

The SNP rs2287886, located close to the AP-1 binding site, was not associated with NPC risk in our study. While this result is consistent with the findings in a North African population [10], it contrasts with a previous report showing that Guangdong individuals carrying the G allele at rs2287886 were at 1.42-fold higher risk of NPC than those with the A allele [16]. This discrepancy may reflect differences in genetic background and linkage disequilibrium between our mostly Zhuang minority subjects and the Cantonese subjects in the Guangdong study.

We observed no significant relationship of rs735240 with risk of NPC based on unadjusted ORs or ORs adjusted for age, sex, and smoking history. However, we found that the frequency of the genotype AA was significantly lower in patients than in controls among subjects negative for EBV-VCA-IgA. Thus, our results suggest that the genotype AA may protect against NPC in individuals negative for EBV-related antibodies. This implies that the SNP rs735240 and EBV infection may interact to influence risk of NPC.

How this interaction occurs is unclear. EBV can infect DC-SIGN-positive cells including monocytes, immature DCs, and some macrophages [16, 18, 19]. Polymorphism in the DC-SIGN gene has already been shown to influence how easily DCs that are infected with cytomegalovirus, a herpesvirus with a structure similar to that of EBV: DCs from individuals with the genotype GG at rs735240 and the genotype AA at rs2287886 (GGAA) express higher levels of DC-SIGN and are more efficiently infected by cytomegalovirus than DCs carrying the AAGG genotype [20]. An analogous situation may exist for EBV, since glycoproteins on the viral surface, which are conserved with those on cytomegalovirus [21], appear to bind to DC-SIGN [16, 22, 23] and allow the virus to enter B cells and epithelial cells [24]. This leads us to propose that the genotype AA at rs735240 protects against NPC by reducing DC-SIGN expression, making it more difficult for EBV to infect DCs and nasopharyngeal epithelial cells.

It is noteworthy in our study that the NPC and control group differed significantly in gender composition and smoking status (Table 1). Although these differences suggested that sex and smoking habit might be confounding factors affecting the results, no significant differences in our following analyses were showed in genotype frequencies at rs2887886 and rs4804803 and rs7252229 and rs735240 between males and females or ever-smokers and never-smokers in both NPC and control groups (detail not shown). Thus, it is unlikely that these SNPs vary with gender or smoking status. In any case, we controlled for sex, smoking status, and age by performing adjustment analyses using logistic regression, thereby eliminating or minimizing any possible confounding effects of these factors.

The results of the present study should be interpreted with caution given its important limitations. One is the relatively small sample, which increases the risk of selection bias. Another is our cross-sectional design, which means we cannot exclude the possibility that some of our controls will go on to develop NPC or other malignant tumors.

## 5. Conclusion

The present study identifies several SNPs in the promoter region of the DC-SIGN gene that may play an important role in NPC pathogenesis. The genotype CC at rs7252229 and the genotype GG at rs4804803 may be associated with elevated NPC risk, while the genotype AA at rs735240 may be associated with decreased NPC risk among individuals negative for EBV-VCA-IgA.

## Figures and Tables

**Table 1:** Clinical characteristic of Chinese patients with NPC and cancer-free controls.

Subgroup	Cases	Controls	*χ* ^2^/*t*	*P*
*N*	477	561		
Gender, *n*			42.121	<0.001^a^
Male	365	322		
Female	112	239		
Age, yr	46.68 ± 11.60	48.17 ± 13.53	1.912	0.056^b^
EBV-VCA-IgA status, *n*			197.811	<0.001^a^
Positive		15		
Negative	314	546		
Smoking history, *n*	163		21.883	<0.001^a^
Yes	163	119		
No	314	442		

^a^Chi-squared test; ^b^two-sample *t*-test. EBV-VCA-IgA: immunoglobulin A against Epstein-Barr virus capsid antigen; NPC: nasopharyngeal carcinoma; OR: odds ratio.

**Table 2:** Frequencies of genotypes at DC-SIGN rs7252229 in NPC patients and healthy controls.

Genotype	Cases	Controls	Unadjusted OR (95%CI)	*P*	Adjusted OR^∗^ (95%CI)	Adjusted *P*	*P*_HWE_
rs7252229							0.307
CC	8	1	1.000		1.000		
GC	65	73	0.111 (0.014–0.914)	0.041	0.076 (0.008–0.690)	0.022	
GG	404	486	0.104 (0.013–0.834)	0.033	0.056 (0.006–0.487)	0.009	
GC + GG	469	559	0.105 (0.013–0.842)	0.034	0.059 (0.007–0.515)	0.010	

^∗^Calculated using multiple logistic regression after controlling for age, sex, smoking history, and EBV infection factors. CI: confidence interval; DC-SIGN: dendritic cell-specific intercellular adhesion molecule 3-grabbing nonintegrin; HWE: Hardy-Weinberg equilibrium; NPC: nasopharyngeal carcinoma; OR: odds ratio.

**Table 3:** Frequencies of genotypes at DC-SIGN rs2287886 in NPC patients and healthy controls.

Genotype	Cases	Controls	Unadjusted OR (95%CI)	*P*	Adjusted OR^∗^ (95%CI)	Adjusted *P*	*P*_HWE_
rs2287886							0.331
AA	236	294	1.000		1.000		
AG	202	229	1.099 (0.851–1.418)	0.469	1.174 (0.778–1.772)	0.444	
GG	38	36	1.315 (0.808–2.140)	0.270	0.819 (0.345–1.94)	0.651	

^∗^Calculated using multiple logistic regression after controlling for age, sex, smoking history, and EBV infection factors. CI: confidence interval; DC-SIGN: dendritic cell-specific intercellular adhesion molecule 3-grabbing nonintegrin; HWE: Hardy-Weinberg equilibrium; NPC: nasopharyngeal carcinoma; OR: odds ratio.

**Table 4:** Frequencies of genotypes at DC-SIGN rs4804803 in NPC patients and healthy controls.

Genotype	Cases	Controls	Unadjusted OR (95%CI)	*P*	Adjusted OR^∗^ (95%CI)	Adjusted *P*	*P*_HWE_
rs4804803							0.533
AA	400	480	1.000		1.000		
AG	69	78	1.062 (0.748–1.506)	0.738	1.269 (0.803–2.005)	0.307	
GG	8	2	4.800 (1.014–22.732)	0.048	9.038 (1.708–47.822)	0.010	
GG + AG	77	80	1.155 (0.822–1.623)	0.406	1.447 (0.934–2.241)	0.098	

^∗^Calculated using multiple logistic regression after controlling for age, sex, smoking history, and EBV-VCA-IgA status. CI: confidence interval; DC-SIGN: dendritic cell-specific intercellular adhesion molecule 3-grabbing nonintegrin; HWE: Hardy-Weinberg equilibrium; NPC: nasopharyngeal carcinoma; OR: odds ratio.

**Table 5:** Frequencies of genotypes at DC-SIGN rs735240 in NPC patients and healthy controls.

Genotype	Cases	Controls	Unadjusted OR (95%CI)	*P*	Adjusted OR^∗^(95%CI)	Adjusted *P*	Adjusted OR^▲^ (95%CI)	Adjusted *P*	*P*_HWE_
rs735240									0.937
AA	24	28	1.000		1.000		1.000		
AG	161	193	0.973 (0.543–1.745)	0.927	1.044 (0.574–1.901)	0.887	2.976 (1.123–7.888)	0.028	
GG	292	339	1.005 (0.570–1.772)	0.986	1.077 (0.601–1.927)	0.804	3.314 (1.274–8.622)	0.014	
AG + GG	453	532	0.993 (0.568–1.738)	0.982	1.065 (0.600–1.891)	0.830	3.191 (1.237–8.230)	0.016	

^∗^Calculated using multiple logistic regression after controlling for age, sex, and smoking history; ^▲^calculated using multiple logistic regression after controlling for age, sex, smoking history, and EBV-VCA-IgA status. CI: confidence interval; DC-SIGN: dendritic cell-specific intercellular adhesion molecule 3-grabbing nonintegrin; HWE: Hardy-Weinberg equilibrium; NPC: nasopharyngeal carcinoma; OR: odds ratio.
